# A Research Agenda for Helminth Diseases of Humans: The Problem of Helminthiases

**DOI:** 10.1371/journal.pntd.0001582

**Published:** 2012-04-24

**Authors:** Sara Lustigman, Roger K. Prichard, Andrea Gazzinelli, Warwick N. Grant, Boakye A. Boatin, James S. McCarthy, María-Gloria Basáñez

**Affiliations:** 1 Laboratory of Molecular Parasitology, Lindsley F. Kimball Research Institute, New York Blood Center, New York, New York, United States of America; 2 Institute of Parasitology, McGill University, Montreal, Canada; 3 Escola de Enfermagem, Universidade Federal de Minas Gerais, Belo Horizonte, Brazil; 4 The Nematode Functional Genomics Laboratory, La Trobe University, Victoria, Australia; 5 Lymphatic Filariasis Support Centre, Department of Parasitology, Noguchi Memorial Institute for Medical Sciences, University of Ghana, Legon, Ghana; 6 Queensland Institute of Medical Research, University of Queensland, Herston, Queensland, Australia; 7 Department of Infectious Disease Epidemiology, School of Public Health, Faculty of Medicine, Imperial College London, London, United Kingdom; Michigan State University, United States of America

## Abstract

A disproportionate burden of helminthiases in human populations occurs in marginalised, low-income, and resource-constrained regions of the world, with over 1 billion people in developing areas of sub-Saharan Africa, Asia, and the Americas infected with one or more helminth species. The morbidity caused by such infections imposes a substantial burden of disease, contributing to a vicious circle of infection, poverty, decreased productivity, and inadequate socioeconomic development. Furthermore, helminth infection accentuates the morbidity of malaria and HIV/AIDS, and impairs vaccine efficacy. Polyparasitism is the norm in these populations, and infections tend to be persistent. Hence, there is a great need to reduce morbidity caused by helminth infections. However, major deficiencies exist in diagnostics and interventions, including vector control, drugs, and vaccines. Overcoming these deficiencies is hampered by major gaps in knowledge of helminth biology and transmission dynamics, platforms from which to help develop such tools. The Disease Reference Group on Helminths Infections (DRG4), established in 2009 by the Special Programme for Research and Training in Tropical Diseases (TDR), was given the mandate to review helminthiases research and identify research priorities and gaps. In this review, we provide an overview of the forces driving the persistence of helminthiases as a public health problem despite the many control initiatives that have been put in place; identify the main obstacles that impede progress towards their control and elimination; and discuss recent advances, opportunities, and challenges for the understanding of the biology, epidemiology, and control of these infections. The helminth infections that will be discussed include: onchocerciasis, lymphatic filariasis, soil-transmitted helminthiases, schistosomiasis, food-borne trematodiases, and taeniasis/cysticercosis.

## Introduction

Since the publication by Norman Stoll in 1947 of “This Wormy World” [1], where the intolerable burden of intestinal nematode infections was highlighted, several global efforts have been made to address the health effects of human parasitism by helminths. Helminths (roundworm and flatworm parasites) are among the most widespread infectious agents of human populations. Today, they disproportionately affect marginalised, low-income, and resource-constrained regions of the world. It is estimated that over 1 billion people in developing regions areas of sub-Saharan Africa (SSA), Asia, and the Americas are infected with one or more species of helminths [2], [3]. The morbidity associated with most of the helminthic diseases we focus on in this report and in the other reviews in this issue are closely linked to poverty; they result from poverty and markedly contribute to further poverty by, among others, impairing agricultural and economic productivity, and they exert a detrimental impact on cognitive development and educational outcomes, thereby hampering socioeconomic development. Moreover, the infections themselves may accentuate the effect of other significant pathogens such as malaria and HIV, and attenuate the response to a range of vaccines.

In response to growing evidence demonstrating the devastating impact of these neglected tropical diseases (NTDs) on the bottom billion of the world population through their effects on health, education, and socioeconomic development, the World Health Assembly (WHA) has adopted several resolutions calling for the control or elimination of these diseases, and for the implementation of a number of large-scale control and elimination programmes. These have been aimed at the parasites themselves and/or the agents (vectors and intermediate hosts) responsible for their transmission. In 1974, WHA resolution WHA27.52 was passed, calling upon the World Health Organization (WHO) to intensify research on major parasitic diseases. This led in 1975 to the creation of the Special Programme for Training and Research in Tropical Diseases (TDR). That year also saw the commencement of antivectorial operations by the Onchocerciasis Control Programme (OCP) in West Africa. In 1993 and 1995, respectively, the Onchocerciasis Elimination Program for the Americas (OEPA) and the African Programme for Onchocerciasis Control (APOC) were initiated. In 1997, resolution WHA50.29 was passed, which urged the WHO and member states to eliminate lymphatic filariasis (LF). This led to the formation of the Global Programme to Eliminate Lymphatic Filariasis (GPELF), which is supported by the Global Alliance to Eliminate Lymphatic Filariasis (GAELF), a public–private partnership that was launched in 2000 to support the GPELF in fundraising, advocacy, communications, resource mobilisation, and programme implementation. In 2001, resolution WHA54.19 was passed, setting the global target of treating by the year 2010 at least 75% of all school-age children at risk of morbidity from soil-transmitted helminthiases (STHs) and schistosomiasis. This resolution led to the establishment of Partners for Parasite Control (PPC) by the WHO. More recently, the global public health community has been invigorated by the articulation of the Millennium Development Goals (MDGs), which are: MDG1: Eradicate extreme poverty and hunger; MDG2: Achieve universal primary education; MDG3: Promote gender equality and empower women; MDG4: Reduce child mortality; MDG5: Improve maternal health; MDG6: Combat HIV/AIDS, malaria and other diseases; MDG7: Ensure environmental sustainability; and MDG8: Develop a global partnership for development [2], [4]. Several new initiatives have been also been established, most notably the Schistosomiasis Control Initiative (SCI) in 2002 and the Global Network for Neglected Tropical Disease Control (GNNTDC) in 2006.

However, despite such WHA/WHO resolutions, the initiatives described above, and the many scientific advances in our understanding of the biology and epidemiology of helminth infections, obstacles remain that challenge the global public health community in their efforts to attain the aims of controlling morbidity and eliminating infection. Some of the identified obstacles include the current scarcity of tools for: 1) updated disease mapping (particularly as interventions progress); 2) new anthelmintics and vaccines that would provide higher levels of control and greater sustainability than currently available to the present control measures; 3) improved, more sensitive diagnostics that are required for specific activities, such as in elimination settings; 4) monitoring the progress of control interventions and quantifying changes in incidence of infection and disease; 5) assessing the efficacy of drug and interventions to control vectors/intermediate hosts (e.g., insecticides) and promptly detecting possible development of resistance to these; 6) determining programme end points (for elimination of the public health burden and/or the infection reservoir) and deciding when interventions could be safely stopped; and 7) implementing post-control surveillance. Research gaps in these and other areas, as well as inclusion of a research and development agenda for human helminthiases for each of the topics covered (interventions, diagnostics, basic biology, mathematical modelling, social and environmental determinants, and capacity building) have been the subject of deliberation by the Disease Reference Group on Helminth Infections (DRG4), established in 2009 by TDR, and will be further discussed in detail in the following reviews within this *PLoS Neglected Tropical Diseases* collection. Box 1 lists the abbreviations used in this paper.

**Box 1. List of Abbreviations**

**ADLA**, acute dermatolymphangioadenitis

**DALY**, disability-adjusted life year

**DRG4**, Disease Reference Group on Helminth Infections

**EST**, expressed sequence tag

**GIS**, geographical information systems

**GAELF**, Global Alliance to Eliminate Lymphatic Filariasis

**GNNTDC**, Global Network for Neglected Tropical Disease Control

**GPELF**, Global Programme to Eliminate Lymphatic Filariasis

**LF**, lymphatic filariasis

**MDGs**, Millennium Development Goals

**MSAT**, mass screen and treat

**NCC**, neurocysticercosis

**NTDs**, neglected tropical diseases

**OCP**, Onchocerciasis Control Programme in West Africa

**M&E**, monitoring and evaluation

**OEPA**, Onchocerciasis Elimination Program for the Americas

**OSD**, onchocercal skin disease

**PPC**, Partners for Parasite Control

**REA**, rapid epidemiological assessment

**RNAi**, RNA interference

**RS**, remote sensing

**SCI**, Schistosomiasis Control Initiative

**STHs**, soil-transmitted helminthiases

**SSA**, sub-Saharan Africa

**TDR**, Special Programme for Research and Training in Tropical Diseases

**WHA**, World Health Assembly

**WHO**, World Health Organization

## Human Helminthiases, Populations at Risk, and Resulting Diseases

Helminth parasites are parasitic worms from the phyla Nematoda (roundworms) and Platyhelminthes (flatworms). Together, they comprise the most common infectious agents of humans in developing countries. The most common helminthiases of humans are those caused by intestinal infection with STHs, namely *Ascarias lumbricoides*, *Trichuris trichiura*, and hookworms (*Necator americanus* and *Ancylostoma duodenale*), followed by schistosomiasis and lymphatic filariasis (LF). In Table 1 the estimated number of people infected (although estimates are given separately, there is a significant amount of co-infection), the burden of disease (in terms of disability-adjusted life years [DALYs]), and the estimated number of annual deaths attributable to each disease, are summarised for each condition. The collective burden of the common helminth diseases rivals that of the main high-mortality conditions such as HIV/AIDS or malaria; 85% of the NTD burden for the poorest 500 million people living in SSA results from helminth infections. Of the 580 million people in Latin America and the Caribbean, 241 million live in areas where at least one of the NTDs is endemic [5], [6]. Since the remit of the series of these review papers is centered on the issues of identifying research priorities for the improvement of helminth control programmes, the infections described below are ordered not in terms of their abundance but in terms of their history of intervention, with the OCP in West Africa (1975–2002) being the first large-scale programme to have been implemented (originally based on vector control).

**Table 1:** The Worldwide Abundance, Burden of Disease, Distribution, and Control/Elimination Programmes of Human Helminthiases.

Infection	Causal Agent	Region with Highest No. Infected	Number Infected (Millions)	DALYs (Millions)	Number of Deaths/Year (Thousands)	Programmes Involved
Onchocerciasis	*Onchocerca volvulus*	SSA	37	1.5^a^	0.05 (in the OCP area)^b^	OCP, APOC, OEPA
Lymphatic filariasis	*Wuchereria bancrofti; Brugia malayi*	India, SEA, SSA	120	5.8	0.4	GPELF
Ascariasis	*Ascaris lumbricoides*	Asia, Africa, LA	1,221–1,472^c^	1.8–10.5^c^	3–60^c^	PPC, DtW, GPELF, SCI
Trichuriasis	*Trichuris trichiura*	Asia, Africa, LA	759–1,050^c^	1.0–6.4^c^	3–10^c^	PPC, DtW, GPELF, SCI
Hookworm infection	*Necator americanus; Ancylostoma duodenale*	Asia, Africa, LA	740–1,300^c^	0.1–22.1^c^	3–65^c^	PPC, DtW, GPELF, SCI
Schistosomiasis	*S. mansoni* *S. haematobium* *S. japonicum*	SSA, LASSAChina, SEA	207	1.7–4.5^c^	15–280^c^	SCI in SSA; national programmes elsewhere
Food-borne trematodiases	*Clonorchis sinensis; Opisthorchis viverinni; Paragonimus* spp.; *Fasciolop-sis buski; Fasciola hepatica*	East Asia	56^d^	0.5–0.9^d^	7^d^	Large-scale control initiatives lacking
Cestode infections: cysticercosis	*Taenia solium*	SSA, Asia, LA	0.4 (LA only)	ND	ND	Large-scale control initiatives are lacking

Modified from references [2], .

a From Remme et al. [10].

b From Little et al. [120].

c From Utzinger and Keiser [14].

d From Fürst et al. [36].

Abbreviations: SSA, sub-Saharan Africa; SEA, Southeast Asia; LA, Latin America; OCP, Onchocerciasis Control Programme in West Africa (1975–2002); APOC, African Programme for Onchocerciasis Control (1995–ongoing); OEPA, Onchocerciasis Elimination Program for the Americas (1993–ongoing); GPELF, Global Program to Eliminate Lymphatic Filariasis (2002–ongoing); PPC, Partners for Parasite Control (2001–ongoing); DtW, Deworm the World (2007–ongoing); SCI, Schistosomiasis Control Initiative (2002–ongoing).

### Onchocerciasis

Onchocerciasis (caused by infection with *Onchocerca volvulus*) affects, according to recent estimates [7], 37 million people in 34 countries and is the second cause of infectious blindness after trachoma, with 99% of the cases in SSA. In Latin America, onchocerciasis has been endemic in six countries (namely, Mexico, Guatemala, Colombia, Ecuador, Venezuela, and Brazil), with 13 focal areas originally described, and 510,000 individuals estimated to have been at risk of infection [7]. However, in eight of these focal areas, there is encouraging evidence of interruption of transmission after the implementation of the regional strategy adopted by the OEPA [5], [6]. Notably, most of these foci are small and circumscribed, with probably not much genetic diversity in the parasite population at any single focus, some (though not all) of the blackfly vectors are less efficient than their African counterparts, and treatment with ivermectin has been more frequent (biannually instead of yearly, and in some localities up to four times per year) and has achieved a high coverage [8].

In onchocerciasis, morbidity is manifested as ocular involvement including blindness, dermal involvement including skin disease and palpable nodules, neuro-hormonal involvement including associations with epilepsy and hypo-sexual dwarfism (Nakalanga syndrome), and lymphatic involvement including lymphadenopathy, hanging groin, and lymphoedema. Although onchocercal ocular disease and blindness are more prominent in African savannah regions, onchodermatitis or onchocercal skin disease (OSD) has a higher prevalence in forest areas, possibly because of differences in parasite strains. The importance of OSD as a contributor to the disease burden of onchocerciasis has been recognised relatively recently [9]. In 1995, the year APOC was implemented (and 25 years after the commencement of the OCP), the burden of disease was estimated to amount to 1.99 million DALYs lost because of onchocerciasis. In 2003, on the basis of updated mapping and treatment coverage data, and assumed decreases in attributable morbidity due to skin and eye disease, the DALYs due to onchocerciasis were re-estimated to have reduced to 1.49 million [10].

### Lymphatic Filariasis

LF is endemic in 83 countries and territories. It is estimated that 1.3 billion people are at risk for developing the disease, with some 120 million people being infected [11]. Over 40 million people are seriously incapacitated and disfigured by the disease. Of these, 95% are infected with *Wuchereria bancrofti*, and the remainder with *Brugia malayi* or *Brugia timori* [12]. The infection, usually acquired in early childhood, causes considerable morbidity and social stigma because of the deformities it produces. LF provokes acute dermatolymphangioadenitis (ADLA) and lymphoedema. Major chronic manifestations include hydrocoele and lymphoedema of limbs, as well as chyluria, lymphoedema of the scrotum, adenopathy, haematuria, and tropical pulmonary eosinophilia. The disease causes permanent and long-term disability, and damages and deforms the limbs, breasts, and genitals, resulting in serious psychosocial consequences. Worldwide, 5 million DALYs are lost annually due to LF [10]. The Southeast Asia region accounts for about 57% of the total global burden. India's economic losses due to LF have been estimated at ∼US$ 1 billion per year [13].

### Soil-Transmitted Helminthiases

The STHs are intestinal nematode infections and are among the most common and persistent parasitic infections worldwide. According to the latest estimates, 1221–1472 million people are infected with roundworm (*A. lumbricoides*), 795–1050 million with whipworm (*T. trichiura*), and 740–1300 million with hookworm (*N. americanus*, *A. duodenale*) [14]. Although not mentioned further in this report, it is worth noting that an unknown, but estimated 30–100 million people are infected with threadworm (*Strongyloides stercoralis*) [15]. The morbidity attributable to this lifelong infection is poorly studied but the serious morbidity and mortality it causes in immuno-suppressed individuals continues to be reported in the medical literature [3], [16]. In Latin America and the Caribbean, STHs are present in all countries with an estimated 26.3 million school-age children at risk of infection. In 13 of the 14 countries in this region, many areas have an infection prevalence higher than 20% [5], [6], [17]. Globally, approximately 300 million people suffer from severe morbidity that results in 10,000–135,000 deaths annually. However, their greatest impact is through the impairment of physical and mental development in children, which ultimately retards educational advancement and economic productivity. The relationship between hookworm infection and anaemia is well recognised, with numerous intervention trials showing a direct effect of cure of infection with reduction in prevalence and intensity of iron deficiency anaemia. However, for *A. lumbricoides* and *T. trichiura* infection, the relationship between infection and specific morbidity measures is less well established. The uncommon, but potentially severe, adverse clinical outcomes of rectal prolapse due to *T. trichiura* infection and bile duct or intestinal obstruction due to *A. lumbricoides* infection are well recognised in the medical literature. However, there is a lack of epidemiological data defining the prevalence of these complications, and relating them to community prevalence and infection intensity [18]–[20]. Less easily measured parameters such as hypoproteinemia have been attributed to heavy infection with these parasites, but likewise there is a dearth of quality epidemiological data. Studies have been undertaken to relate school, cognitive, and athletic performance to infection, largely through chemotherapy interventions. While there appears to be some effect, distinguishing this from other coexisting confounding variables such as micro- and macronutrient deficiency is not straightforward.

### Schistosomiasis

Schistosomiasis is endemic in 76 countries and territories in the tropics and subtropics. *Schistosoma mansoni* is endemic in 54 countries and *S. haematobium* in 55 [21]. Infection by *S. japonicum* remains an important public health burden in the Philippines, China, and parts of Indonesia, despite continued efforts by ongoing control programmes [22]. Worldwide, almost 800 million individuals are at risk; about 200 million people are estimated to be infected, and over half of these have various degrees of morbidity [23]–[25]. Of the 200 million people infected with *Schistosoma* spp. in the world, 160 live in SSA, where approximately 110 million are infected with *S. haematobium* [26]. Schistosomiasis causes 15,000–280,000 deaths annually in SSA alone [26] and severe disability in approximately 20 million people. In its chronic stage, the disease is associated with liver and bladder cancer.

Infection with *S. haematobium* is particularly burdensome, causing a large number of cases of hydronephrosis, renal failure, and bladder cancer. Women with urinary/genital schistosomiasis are at an increased risk of acquiring HIV infection [27]. Over 80% of the schistosomiasis burden is concentrated in SSA. In Latin America and the Caribbean, *S. mansoni* infection is prevalent in four countries: Brazil, Saint Lucia, Suriname, and Venezuela, with 25 million people at risk of infection and 1–3 million people infected. The figures presented in Table 1 (corresponding to the Global Burden of Disease estimates for 2000 and updated in 2004) are thought to considerably underestimate the true burden of schistosomiasis [28], [29]. As for almost all helminth infections, schistosome infection is not equivalent to schistosomiasis disease. Likewise, there is a paucity of validated direct and indirect indicators of schistosome-related morbidity [30]–[33]. In urinary schistosomiasis, macroscopic haematuria is an obvious early sign of morbidity. However, for *S. mansoni* and *S. japonicum*, assessment of morbidity is more difficult. Furthermore, few, if any, of the clinical manifestations of schistosomiasis are specific and overlap with other causes, including other helminth infections, malaria, and viral hepatitis, which often are co-endemic with schistosomiasis.

### Food-Borne Trematodiases

Food-borne trematodiases, including liver flukes (*Opisthorchis viverrini*, *Op. felineus*, and *Clonorchis sinensis*) and lung flukes (*Paragonimus* spp.), remain important public health problems, particularly in Asia. Chronic infections with *Op. viverrini* and *C. sinensis* have long been associated with cholangiocarcinoma (bile duct cancer). *C. sinensis* is widespread in China, Korea, and Vietnam, while *Op. viverrini* is endemic in Southeast Asia, including Thailand, Lao People's Democratic Republic (Lao PDR), Cambodia, and central Vietnam. Recent reports suggest that about 35 million people globally are infected with *C. sinensis*, with up to 15 million human infections in China alone and another 10 million individuals infected with *Op. viverrini* in Thailand and Lao PDR. In a recent review it was estimated that 80 million people in Thailand, Lao PDR, Cambodia, Vietnam, and Eastern Europe are at risk for infection with *Op. viverrini* and *Op. felineus*. Over 45 million people are infected by both liver flukes *Op. viverrini* and *C. sinensis*. More than 600 million people, mainly in Asia, including China, Korea, Taiwan, and Vietnam, are at risk of infection [34]. The infections are associated with hepatobiliary diseases including hepatomegaly, cholangitis, fibrosis of the periportal system, cholecystitis, gallstone disease, and, importantly, are major precipitants of cholangiocarcinoma. The liver fluke–endemic area of Khon Kaen in northeast Thailand has reported the highest incidence of this liver cancer in the world [35]. Indeed, liver and bile duct cancers, end-stage consequences of liver fluke disease, rank number five in Thai males and number six in females among all diseases in terms of DALYs in 2005. About 56.2 million people were infected with food-borne trematodes in 2005, 7.9 million had severe sequelae, and 7,158 died, mostly from cholangiocarcinoma and cerebral infection. Taken together, the global burden of food-borne trematodiasis was estimated at 665,352 DALYs (ranging from 479,496 to 859,051) [36].

### Taeniasis/Cysticercosis

Human infections with the cestode parasite *Taenia solium* are endemic in Latin America, most parts of Asia (including China and the Indian subcontinent), Eastern Europe, and most of Africa. Imported cases occur in most developed countries due to immigration from and tourism to endemic regions. *T. solium* infection, which causes intestinal taeniasis and tissue cysticercosis, represent two different life stages and clinical entities in the human host. Intestinal taeniasis with the adult tapeworm (when humans act as definitive hosts) causes low morbidity by itself, but represents the sole source of the more pathogenic tissue infection, designated cysticercosis, that affects both humans and pigs. Cysticercosis, the infection with the larval stage of the parasite or cysticercus (when humans act as “intermediate hosts”), is a major cause of seizure disorders worldwide (human neurocysticercosis or NCC), and also causes economic losses due to infected pork (porcine cysticercosis). The most consistent indicator of the prevalence of neurocysticercosis is that of seizure disorders. In several endemic areas of Latin America, the attributed fraction for NCC is around 30% of all seizure disorders, with this estimate also being consistent among people with epilepsy worldwide [37]. Subarachnoid NCC is also associated with intracranial hypertension and mortality.

Notably, the prevalence of NCC worldwide remains still unknown. However, initiatives are currently underway to determine the burden of NCC in endemic countries of Africa, Asia, and Latin America [37]–[39] as well as in industrialised countries such as the United States where it is becoming a better recognised and possibly an increasing problem, the latter because of immigration of tapeworm carriers from endemic areas. As the estimate for the proportion of NCC among people with epilepsy is very robust [37], it could be used, in conjunction with estimates of the prevalence and incidence of epilepsy, to estimate this component of the burden of NCC in endemic areas.

## Distribution and Co-Occurrence

**Figure 1:**
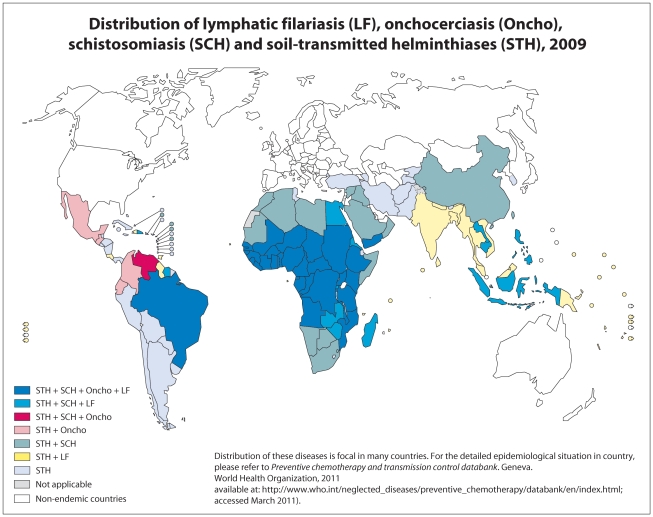
Geographical distribution of co-infections with helminth infections, 2009. The helminth infections include: lymphatic filariasis (LF), onchocerciasis (Oncho), schistosomiasis (SCH), and soil-transmitted helminthiases (STH). The different colors represent the following co-infections: STH+SCH+Oncho+LF; STH+SCH+LF; STH+SCH+Oncho; STH+Oncho; STH+SCH; STH+LF; and only STH. The information is based on reference [119].

Epidemiologically, human helminthiases are characterised by long-lasting infection with one or, more often, more than one of the helminth species referred to above. This phenomenon, known as polyparasitism, is the result of commonalities in ecological and environmental requirements, infection routes, host exposures, and susceptibility, as well as behavioural, sociological, and economic factors that enable co-occurrence of a multiplicity of parasite–host systems in time and space. In Figure 1, the geographical distribution of co-occurrence of helminth species (LF, onchocerciasis, schistosomiasis, and STHs) at country level is presented.

As studies have demonstrated that individuals infected with multiple helminth species may also have the most intense infections [40]–[44], polyparasitism may have a greater impact on morbidity than the sum of single-species infections. In addition, multiple species infections may increase susceptibility to other infections, such as malaria or HIV [45], [46], particularly given the often detrimental immunomodulatory effect of helminth infections. Consequently, efforts have been made to better understand the consequences of the co-existence of parasites within the same host on the immunological responses to each species and, more importantly, whether such interactions affect resistance, susceptibility, or clinical outcome [47]. Co-infections are also shifting some of the prevention and control measures of helminth infections from single-drug treatments to integrated approaches that can simultaneously target as many as four of the helminth NTDs, including onchocerciasis, LF, STHs, and schistosomiasis—a mission undertaken by the GNNTDC and the WHO [2], [48].

## Advances, Opportunities, and Challenges for the Control and Elimination of Human Helminthiases

Improvement of environmental conditions, increased hygiene, access to clean and potable water, better housing, and sustained socioeconomic development have been demonstrated to be essential for the elimination of helminth infections (the reason many of these infections, previously prevalent in presently industrialised countries, are no longer endemic in such areas). Despite this, treatment of populations with anthelmintics in the modality of mass drug administration (MDA), referred to as preventive chemotherapy via community-based distribution systems and through schools, has become the predominant tool for helminthiasis control in developing countries, either as targeted treatment (to particular occupational or age groups), or among whole communities, since they appear to be among the most cost-effective global public health control measures [49]–[51]. Not only does MDA obviate the costly requirement for mass screening and diagnosis, but also drug donation programmes have been initiated by highly visible pharmaceutical companies (such as the Mectizan Donation Program of Merck & Co. for onchocerciasis and LF control/elimination with ivermectin, in coordination with albendazole donation by GlaxoSmithKline for LF and STHs [52]). Additionally, patents have expired for some compounds, making generic preparations (such as praziquantel for schistosomiasis, or diethylcarbamazine for LF) highly affordable. The anthelmintic drugs thus available to the control programmes are safe for mass treatment of human populations and moderately to highly (albeit variably) efficacious (see review by Prichard et al. in this issue for a summary and discussion on anthelmintic efficacy [53]).

MDA is being assisted by global partnerships, including the aforementioned donations from pharmaceutical companies of anthelmintic drugs and financial support from foundations, governments, United Nations agencies, companies, and individuals. Efforts to integrate various MDA programmes may bring logistic benefits to intervention programmes. However, it should also be recognised that very little funding is available to support operational research that is essential for such interventions to remain sustainable and bring long-lasting benefits. Unfortunately, much of the effort is, at present, directed at short-term objectives.

Mass chemotherapy as a control strategy has its own challenges, among which are those of optimising community involvement and participation. The recognition, acceptance, and commitment to play the role that is expected of the communities are factors crucial to achieving sustainable control programmes. Although global funding for these programmes has increased markedly in recent years, “donor fatigue” may set in as a major obstacle to sustained funding if initial successes (which are invariably largest at the beginning), are not maintained. The arsenal of available drugs for MDA is limited, as most of them were developed originally for parasites of veterinary importance in markets of middle- to high-income economies,with very little or no development programmes for new drugs specifically targeting human helminthiases. This makes the existing control programmes highly vulnerable to the possible development and spread of anthelmintic resistance. It is also clear that the long-term control and eradication of these helminth diseases will require improvements in sanitation and hygiene, improved socioeconomic development, and environmental sustainability.

Increasing human population size, changes in demographic patterns and activities, and changing agriculture and irrigation practices are also altering the environment at an unprecedented scale, further increasing the risk of STH and zoonotic helminthiases [54], [55]. How these activities will play out in the long term, as well as what will be the effects of climate change on the distribution and incidence of helminth infections, remains poorly understood. Thus, whereas helminth diseases are often thought of as chronic and ancient scourges of humanity, some may become re-emerging diseases as new outbreaks are reported in response to environmental and socio-political changes, migration, travel, forced human displacement, and clean water shortages.

Control of *S. japonicum* and most cestode infections in humans is also complicated by the fact that they constitute zoonotic infections. For example, other animals, e.g., cattle, buffalo, rodents, dogs, sheep, and pigs can act as reservoirs for human transmission of *S. japonicum*. A more integrated strategy for control of this species of schistosome is therefore required. A pilot study for such an integrated control strategy for schistosomiasis has been undertaken in China using a multi-pronged approach, including health education, access to clean water and adequate sanitation, mechanisation of agriculture, and fencing of water buffaloes, along with chemotherapy [56]–[58]. In the case of cestode infections, and despite the proven benefit of chemotherapy with praziquantel and possibly albendazole for patients with parenchymal NCC [59], [60], there is a need for improved drugs and/or studies with drug combination regimens and dosages to evaluate their efficacy [61].

Millions of doses of anthelmintics have been administered to patently infected and exposed individuals in endemic areas, in some cases for prolonged periods, and have undoubtedly yielded health benefits for the treated populations. However, helminth infections persist in their host populations, and are resilient to control interventions (a consequence, in part, of their population biology). Understanding the biological, environmental, and social determinants of such persistence, as well as the driving forces of new emerging and re-emerging public health challenges, is crucial to steering research, harnessing the potential of new scientific advancements, and engaging stakeholders to achieve the MDGs.

Reviews of the progress on the implementation of the MDGs indicate that the attainment of the goals has been slow, in particularly those related to health, namely, MDG4, reduce child mortality; MDG5, improve maternal health; and MDG6, combat HIV/AIDS, malaria and other diseases. This is particularly worrying for many African countries. This slow progress is not consistent with the actual increase in resources for health research worldwide. However, it is important to pay attention to the fact that only 5% of global research spending is estimated to actually be applied to the needs of low- and middle-income countries, and a miniscule fraction of this to the neglected helminth diseases. In fact, though MDG6 specifically mentions HIV/AIDS and malaria as critical targets for sustainable poverty reduction by the year 2015, it merely alludes to chronic parasitic worm infections as “other diseases” [2]. Because these diseases prevent the achievement of the first six MDGs, their control with cost-effective interventions could be the basis for long-term economic growth and development [2], [4].

Although the disability among the bottom billion that results from NTDs, including the helminth diseases, is enormous, the NTDs have not received nearly the same attention as three of the highest mortality-causing infectious diseases, HIV/AIDS, malaria, and tuberculosis [62]. This is likely because they are not perceived as major causes of premature death. However, onchocerciasis can cause visual impairment, blindness, and excess mortality both of the blind and of heavily infected yet sighted individuals; LF can cause major body deformation and impaired function, reducing the ability of people to work and look after themselves and others; STHs and schistosomiasis can markedly reduce the growth and development of children, including cognitive ability throughout life, as well as increasing child mortality. Hookworm infection can cause anaemia and impact on maternal health and neonatal mortality. In pregnant women, the same infections may result in premature birth, low birth weight, and increased maternal morbidity and mortality. Liver fluke infections can cause major liver pathology, including hepatic cancer, while NCC is a major cause of seizure disorders and other forms of neurological disease that can increase the level of poverty. Box 2 summarises some of the driving forces that maintain helminthiases as a challenge to the global public health community. They are discussed more fully below.

**Box 2. Factors Driving the Persistence and Re-Emergence of Helminth Infections of Humans**

- A disproportionate burden of helminthiases in human populations occurs in marginalised, low-income, and resource-constrained regions of the world that are extremely poor and live with inadequate sanitation, which contributes to a vicious circle of infection, poverty, decreased productivity, and inadequate socioeconomic development
- Polyparasitism; human helminthiases are characterised by long-lasting infection with one or, more often, more than one helminth and with other diseases
- Free-living stages of some STHs are very resistant to environmental degradation and can persist in soil for years
- Parasite populations are strongly regulated within their hosts (including definitive, intermediate, vector, and snail hosts), which makes them highly stable and often resilient to control interventions; therefore, premature cessation of interventions may lead to re-emergence and eventually restoration of the parasite population to baseline levels
- The control programmes rely heavily on anthelmintic treatment and in many cases they depend on only one drug. This makes the programmes vulnerable to the possible development of anthelmintic resistance
- Optimum treatment coverage with MDA is required for the success of control and elimination programmes for helminthic infections. It also depends on community involvement and participation, which needs to be optimised and further encouraged
- Lack of highly effective tools, e.g., macrofilaricides or vaccines, which can remove a high proportion of an existing infection
- Increasing human population size and activities, and changing agriculture and irrigation practices are also altering the environment at an unprecedented scale, further increasing the risk of zoonotic helminthiases
- Technical limitations of available diagnostic methods for helminthiases impose significant constraints on current initiatives to understand the epidemiology and control of these infections
- A major obstacle to the implementation of cost-effective control is the lack of accurate descriptions of the geographical distribution of infection and co-infection
- Current diagnosis tools do not adequately identify true infection status, parasite load, and do not account for other confounding or interacting infections
- The distribution and burden of helminth infections are not merely a reflection of geographical and ecological circumstances, but also a reflection of the level of political commitment and investment in human and financial resources by national governments for the prevention and control of helminthiases
- A multi-disease, inter-programmatic, and inter-sectorial approach is not always taken wherever scientifically, logistically, and economically possible in order to successfully control helminthiases
- Understandably, priority is given to “applied” or “operational” research at the expense of basic research; however, a continuous effort to improve and update knowledge of helminth fundamental biology is likely to yield improved intervention and control tools

Prof. David Molyneux, one of the leading advocates for NTDs and helminthic control programmes, has said “My position has always been that if you are going to do anything about the MDGs, which have the overall objective of taking people out of poverty, then you had better do something about the diseases which affect the most people rather than those that affect the minority” [63]. However, to control the high-burden NTDs, including helminthiases, in low- and middle-income countries and thus help achieve the MDGs, a great deal of investment from both international and national funding bodies will be required in order to develop the facilities and the capabilities of scientists who can drive research aimed at developing more effective tools and strategies to fight infectious diseases of poverty [4]. Global financing mechanisms for NTDs should take into consideration that disease control programmes must be nationally owned, embedded into national health plans, and backed by political commitment [4].

Another major issue that affects significantly the capacity of several nations to reach the MDGs is the lack of trained human resources, or the inability to retain those individuals who have been trained, due to an inadequate environment for them to tackle health problems within a research culture. Although some countries are in the process of overcoming these difficulties, and several high-level meetings on health research have called for action on this respect (Mexico City, Abuja, Accra, Algiers, and more recently Bamako), a clear commitment has not yet been made by all participating nations. In Bamako, during the Call to Action 2009 meeting, all stakeholders were urged to “promote and share the discovery and development of, and access to, products and technologies addressing neglected and emerging diseases which disproportionately affect low- and middle-income countries”. In countries where NTDs are endemic, different levels of commitment toward their resolution, however, occur. A more detailed discussion regarding these issues is presented in the review entitled “A Research Agenda for Helminth Diseases of Humans: Health Research and Capacity Building in Disease-Endemic Countries for Helminthiases Control” [64].

## Factors Driving the Persistence and Re-Emergence of Helminth Infections of Humans

### Parasite Population Biology and Human Host Sociological Factors

Infection prevalence at any time-point may be high and this, in addition to reflecting a high intensity of transmission, also results from infections which are of long duration: parasites may have a long life span, hosts do not recover and do not mount an effective infection-clearing immune response, and they are exposed to repeated infection throughout their lives.

Further, aggregation of infection, whereby a minority of individuals harbour very heavy infection but the majority of the population harbour light or moderate infection, is a major factor to consider in epidemiological studies and chemotherapy efficacy trials, as well as in efforts to reduce prevalence, if, for example, “wormy” individuals are somehow missed in drug treatment programmes.

Among the biological determinants of persisting infection is the fact that parasite populations are strongly regulated within their hosts, including definitive and intermediate hosts (arthropod vectors, snail intermediate hosts, etc.), which makes them highly stable and often resilient to control interventions. Therefore, premature cessation of interventions may lead to re-emergence and eventually restoration of the parasite population to baseline levels. Among the sociological factors contributing to this stability are those that link infection, and particularly heavy infection, with chronic and long-lasting morbidity, disability, insidious and irreversible effects on health, poor school performance, impaired ability to work, low economic productivity, and premature death. This perpetuates the vicious circle that links helminthiases to poverty, lack of sanitation, poor hygiene, and marginalisation.

All of the above point towards the need to shift the intervention paradigm from single to multi-disease, and to integrated approaches to achieve sustained control of helminth infection. There is also a need to develop indicators capable of capturing meaningful impacts of multiple interventions such as reduced anaemia, improved school attendance, and increased economic productivity, as well as a need to evaluate the cost-effectiveness of such interventions.

### Chemotherapeutic Factors

The majority of the control programmes listed in Table 1 relies heavily on anthelmintic treatment. Overall they have achieved good results with regards to effecting reductions on the prevalence and intensity of infection and morbidity in some endemic areas [65]–[68], and interruption of transmission for some infections, leading towards local elimination, has been reported for some foci [69]–[72] and even at the country level. Between 2000 and 2007, the GPELF prevented LF disease in an estimated 6.6 million newborns who would otherwise have acquired LF, thus averting in their lifetimes nearly 1.4 million cases of hydrocoele, 800,000 cases of lymphoedema, and 4.4 million cases of subclinical disease [11], and achieving considerable economic benefits [73]. Examples of elimination of transmission at a country level include the achievements of the OCP in West Africa that, after 30 years of concentrated efforts, prevented 600,000 new cases of onchocerciasis and protected a total of 18 million villagers from the threat of contracting river blindness [10]. Elimination of LF as a public health problem has been successful in China and Korea [74]–[76], proving that elimination of LF is possible given the necessary levels of political support, adequate funding, public commitment, and improvement of general socioeconomic conditions [76]. In programmes where cohorts have been followed longitudinally from the beginning of the interventions, reductions in the incidence of infection and/or morbidity have been documented [77], [78].

One reason for the heavy reliance on chemotherapy is because for some programmes the drugs are donated by pharmaceutical companies, or their cost has become affordable [2]. In this setting, such interventions have been termed “a rapid-impact package” because the impact of anthelmintic treatment on parasite populations is proportionally largest at the beginning of the intervention. However, the long-term sustainability of the benefits accrued depends critically on altering the environmental components that facilitate transmission. Large-scale elimination of the infection reservoir will depend on improving sanitation and drainage, providing access to clean water, disposing adequately of excreta and solid waste, promoting access to health services for diagnosis and treatment, and facilitating adequate housing and health education [79], [80]. Anthelmintic treatment should therefore be seen as a necessary, but not sufficient, condition towards breaking the vicious circle between helminth infection, ill-health, and chronic poverty.

Anthelmintic treatment can be distributed using a variety of treatment strategies, depending on the level of infection endemicity, the overall aim of the control programme (elimination of the public health burden or of the infection reservoir), the population groups that exhibit the highest infection levels, and the relationship between infection and disease sequelae (for morbidity control). Treatment can be aimed at particular occupational or age groups (such as school-age children) most at risk of heavy infection and severe morbidity. This is the basis for school-based health programmes aimed at deworming children of STHs and schistosomiasis. In this target population, treatment is administered to all individuals regardless of their infection status. In areas where community parasite burden is substantial, community (mass) treatment is recommended. Other strategies include mass screen and treat (MSAT) (targeting selective treatment to those with patent and detectable infection), or treating individual cases in clinical as opposed to community settings. The adoption of such treatment modalities may also depend on the stage of the control programme, with MDA implemented at its commencement, and selective treatment in mopping-up phases. In programmes where the aim is to eliminate the infection reservoir, the approach adopted has generally been to treat the largest number of people at the highest possible coverage for as long as autochthonous transmission persists. However, this approach imposes strong selection pressure upon parasite genomes, affecting genetic diversity and favouring emergence of drug resistant strains. This could lead to resurgence of infection in areas previously under control.

Optimum treatment coverage with MDA is required for the success of control and elimination programmes for helminthic infections. Mathematical models predict, and experience confirms, that population coverage is a key determinant of the success of such programmes and that there remains a need to evaluate the compliance of the population participating in such programmes over the years. The use of the term “therapeutic coverage” implies that the persons who receive the drugs are actually taking them (i.e., they are compliant), but in several countries, most notably India, a gap between coverage and compliance has been observed in the case of LF [81], [82]. The contribution of non-compliant persons to transmission is unknown, but systematic non-compliance may represent a potential threat for helminth control or elimination. Furthermore, the sustainability of the required long-term treatment programmes also raises issues of compliance related to possible population fatigue, waning interest of community drug distributors, and the necessary continuing funding support from external donors, as the original funds which initiated the treatment-based control programmes may be time-limited.

### Factors Associated with Current Ability to Diagnose Helminth Infections in Individuals, Communities, and Larger Spatial Scales

The technical limitations of available diagnostic methods for helminthiases impose significant constraints on current initiatives to control these infections. Appropriate diagnostic methodologies are required for: disease mapping to guide initiation of interventions; case-based diagnosis; and monitoring and evaluation (M&E) of intervention programmes, particularly with the possible threat of failure of the interventions due to technical reasons and/or the development of drug resistance in the parasite and insecticide resistance in vectors. An understanding of all these factors will be important for determining the appropriate timing of cessation of interventions in elimination settings, confirming interruption of transmission, and learning what methods for epidemiological surveillance in post-control areas are needed to monitor resurgence or reinvasion of infections in areas where elimination may have been certified.

For each of these activities, the technical requirements for diagnostic tests are likely to be different, and may represent different technical challenges. Furthermore, for each of the helminth species considered here, the effect of the biology of the parasite (life cycle, accessibility to parasitological diagnosis, body fluid appropriate for sampling, role of and need to sample vector or intermediate hosts), and the biology of the parasite–host system (including age profiles of infection prevalence and intensity) on our ability to diagnose infection status appropriately differs. In addition, intensity of infection is a critical determinant of morbidity and disease burden, and as described above, also critical for the stability of the host–parasite relationship, contribution to density-dependent, regulatory mechanisms of parasite population abundance, and contribution to transmission. Yet, with few exceptions, this critical parameter is difficult to quantify accurately with present tools, or not at all quantified in operational settings.

Not only is the diagnosis of individual infections important, but also is the understanding of their spatial distribution within countries, communities, and in individuals in which they may co-occur. The effectiveness of large-scale integrated programmes for the control of NTDs in general, and of helminth diseases in particular, will depend on the geographical overlap between the different NTDs. However, despite being co-endemic in particular countries [83], different NTDs may, in certain settings, have limited geographical overlap at sub-national scales, necessitating a more geographically targeted approach for integrated NTD control [79], [84]. A major obstacle to the implementation of cost-effective control is the lack of accurate descriptions of the geographical distribution of infection. In recent years, considerable progress has been made in the use of geographical information systems (GIS) and remote sensing (RS) to better understand helminth ecology and epidemiology, and to develop low-cost and minimally invasive ways to identify target populations for treatment such as the development of methods for rapid epidemiological assessment (REA) of infection and morbidity [85], [86]. A significant constraint on such activities is the paucity of accurate information on the distribution of infection prevalence and intensity to build such maps. In China and Africa, predictive maps have been prepared to identify risk areas and help governments and health services to plan control strategies. However, the potential of NTD mapping has been less exploited in Latin America (but see [87] for REA methods and [88] for GIS and onchocerciasis in the Amazonian focus [89]). Thus, research is required to determine the optimal strategy for rapidly and simultaneously assessing a number of NTDs so that more effective implementation of integrated approaches for disease control can take place. Reliable and updated maps of helminth infection distributions are essential to design control strategies to target those populations in greatest need; this current lack constitutes a driving force of persisting control challenges. A principal advantage of GIS platforms is that they facilitate the regular updating of information, and provide a ready basis for analysis and statistical modelling of spatial distributions [90].

### Factors Associated with the Environmental and Social Ecology of Human Helminthiases

The distribution and burden of helminth infections are not merely a reflection of geographical and ecological circumstances, but also a reflection of the level of political commitment and investment in resources (human, financial) by national governments for the prevention and control of helminthiases in these vulnerable populations and the environment they inhabit. Progress has been slow in preventing, controlling, or eliminating these diseases in some countries. Few health care systems can guarantee full access to and delivery of the essential medicines for all patients and populations at risk. Most countries have not yet fully taken advantage of the new tools and protocols available for prevention, control, and elimination. In order to develop an operative strategy for the prevention and control of NTDs, there is a need to organise public health resources.

For the infections that can be controlled by mass chemotherapy (STHs, schistosomiasis), but not readily eliminated, early and intensified case detection and management can be pursued. However, although in some settings MSAT strategies have been implemented (e.g., Brazil), more often than not, after some initial assessment of prevalence and intensity, MDA is implemented; a strategy that does not target infected individuals only. Normally, clinical services are not required to implement such treatment as it can be delivered by community distributors. The complex and substantial epidemiological pattern of these infections in endemic countries makes elimination challenging, particularly in areas of high endemicity [91]. In specific circumstances, however, the feasibility of achieving this goal is being considered (e.g., schistosomiasis in some Caribbean countries and low transmission areas [92], and some isolated (island) foci in Africa such as on Unguja, Zanzibar, http://score.uga.edu/Elimination.html).

Surveillance for NTDs should be the responsibility of national and local health authorities, but there are challenges regarding the availability of appropriate tools for this task (see review by McCarthy et al. in this issue [93]). Moreover, given the reality that most health care systems do not yet prioritise surveillance of NTDs, outreach is needed to extend to and involve cooperating health services, health professionals, environmental health officers, and communities that can organise themselves for surveillance.

At a regional, supra-national level, a ranking order of the relative importance of NTDs would be difficult to propose. Rankings could be made based on criteria such as presence of global or regional mandates for elimination, magnitude of geographical extension, trends in distribution, and estimated burden of disease. However, disease burden in particular is challenging to establish due to lack of reliable data for many NTDs, with factors such as incidence, prevalence, intensity, association with morbidity and sequelae, distribution in afflicted populations, and reliable demographic denominators of populations at risk are all of relevance. In any particular country or sub-region, it will be necessary to prioritise which NTDs will be the most important, based on updated knowledge on local prevalence, disease burden, at-risk populations, geographical distribution, and the commitment of governments to equity in health care (coverage and health services goals). In the strategic lines of action to be developed under this framework, a multi-disease, inter-programmatic, and inter-sectorial approach should be taken wherever scientifically, logistically, and economically possible. It should actively seek community involvement, with the aim of increasing local empowerment. In this way, the focus of implementation should be through work in partnership with multiple sectors and multiple health programmes. Failure to do so is also a driving force of persisting, emerging, and re-emerging helminth infections and the public health challenges they pose.

### Factors Associated with Research Gaps and Overlooking the Role of Basic Helminth Research

One of the main factors associated with the existence of research gaps in basic helminth biology is the understandable priority given to applied activities at the expense of basic research. “Understandable” because of the imperative to better control helminth infections, or to at least relieve the morbidity associated with these infections. The current efforts, based primarily on MDA, have been effective in some cases (e.g., onchocerciasis, LF), but this success has further eroded support for fundamental research. However, MDA is potentially vulnerable to the development of drug resistance in those cases where chemotherapy has had a major impact on parasite prevalence and transmission (and hence exerts a strong selective pressure), and has suffered from the lack of suitable, safe, effective, and affordable drugs in other areas. For example, although the macrocyclic lactone ivermectin has proven effective in the interruption of onchocerciasis transmission in specific epidemiologic settings, no safe and effective macrofilaricide (against adult worms or macrofilariae) exists. Likewise, schistosome control depends critically on the sustained clinical efficacy of praziquantel; the chemotherapy deployed to control many helminth infections in a MDA setting is unsatisfactory, due to the lack of safe and effective drugs amenable to single-dose therapy. Furthermore, there is the hypothetical potential for unintended consequences of MDA stemming from the poorly understood dynamics of host–parasite interactions and interactions between species parasitising the same hosts, and the changes that altering parasite infection intensity and prevalence of some species may have on those interactions.

What is needed is a balanced investment in basic research in parallel to the operational activities that are being rolled out; the dearth of funding available for basic research [94] undermines the further improvement and future sustainability of the control efforts already deployed and the consolidation of the gains that have been achieved. What areas of basic research are likely to yield improved control tools? Comparison of helminths with malaria may be instructive in this context: genomics has transformed malaria research over the past decade, reinvigorating drug and vaccine development, and enabling the development of better parameterised mathematical models that may be used to inform control and elimination efforts. A similar investment in helminth genomics, most particularly in the development of functional tools with which genome sequences can be probed and mined, could catalyse a similar transformation of helminth research.

A major limitation on this front is the limited availability of annotated genome sequences. With the advent of novel rapid and inexpensive genome sequencing technologies, the entire genomes of some selected helminths of medical importance have been successfully sequenced. However, there is a lack of bioinformatic tools, power, and reference genomes to accurately annotate them. The genomes of *B. malayi*, *S. mansoni*, and S. *japonicum* have been partially annotated, and a low-cost alternative for genome sequencing, expressed sequence tag (EST), has been produced for many helminths, which provide a glimpse of the transcriptome of these species [95]–[100]. However, the complexity of helminth life cycles, including the various developmental transitions they undergo, makes it a daunting task to understand these transcriptome profiles and, more importantly, the biological role of the genes identified in these studies [101]. Functional genomic tools such as RNA interference (RNAi) technology has proved to be useful in deciphering the role of schistosome encoded proteins [102]–[105]. In contrast, RNAi technology has been a largely unsuccessful tool for functional analysis in parasitic nematodes [106], [107]. The exact reasons for this are still unclear, but is has been suggested that some essential components of the RNAi pathway are missing in some species of parasitic nematodes [98], [102].

Numerous studies have indicated that helminth-secreted proteins, glycoproteins, and lipid-based molecules can interfere with various host immune responses, ultimately leading to the generation of an anti-inflammatory environment favourable for the parasites' survival [108]–[110]. Some of the processes affected include the development of allergic responses [111], [112] and interference with host cytokine regulation and signal transduction networks [113], [114]. These findings highlight the complexity of the biology of helminths with respect to the human/non-human (definitive) hosts, and are further complicated by the effects of polyparasitism, either by other helminths, protozoan parasites, or bacterial or viral infections. In addition, the importance of *Wolbachia*, the bacterial endosymbiont of filarial nematodes, in *B. malayi*, *W. bancrofti*, and *O. volvulus* has yet to be fully understood with respect to the ultimate effects on host–parasite interactions [115]. *Wolbachia* undoubtedly confer a survival advantage to *B. malayi* and other filarial nematodes that enhances their parasitic potential [116], [117]. Further exploration of this interaction may lead to development of novel methods of eliminating filarial infections in host populations [118].

Despite the significant advances made in genomic, proteomic, and transcriptomic profiles of helminths, these “omics” are still in their early developmental stages. The influx of bioinformatic knowledge needs to be reconciled with novel research methodologies for functional analysis of genes and the proteins they encode. Since most countries afflicted by helminths are still classified as developing, there is a divide between the available resources for the application of basic research and what is available to scientists in the endemic countries. Thus, the urgency required to explore the incoming wealth of bioinformatic knowledge is skewed towards the few research institutes in Western and developed countries that have funding which normally has imposed priorities tailored towards the funding agency's interests and not necessarily the actual needs of affected the population and countries [2].

## Conclusion

The increasing acknowledgement of the burden imposed by helminthiases, particularly since the last quarter of the 20th century, has led to the implementation of large-scale control and elimination programmes. Despite much advancement in controlling these diseases, obstacles remain that challenge the global public health community. There is a clear need for an innovative research framework that is thoroughly integrated with the programmes, holistic in approach, collaborative and global in perspective, and that assesses current understanding, identifies gaps in knowledge, and seizes opportunities to address specific needs and move the helminth control agenda forward. Although millions of doses of anthelmintics have been administered to patently infected and exposed individuals in endemic areas, in some cases for prolonged periods, helminth infections still persist in their host populations and they appear to be resilient to control interventions (a consequence, in part, of their population biology). Moreover, helminth diseases, which are often considered as chronic and ancient scourges of humanity, may become re-emerging diseases as new outbreaks are reported in response to environmental, demographic, and socio-political changes, migration, travel, forced human displacement, and shortages of clean water. Understanding the biological, environmental, and social determinants of such persistence, as well as the driving forces of these emerging and re-emerging public health challenges, is crucial to steering the needed research and to harness the potential of new scientific advancements that can be translated into improved or novel intervention tools. It is important to remember that a large-scale elimination of the infection reservoir will also depend on improving sanitation, providing access to clean water, disposing adequately of excreta and solid waste, promoting access to health services for diagnosis and treatment, and facilitating adequate housing and health education. Box 3 lists some of the research gaps identified in five major research themes, including interventions, diagnostics, basic biology, mathematical modelling, social and environmental determinants, and capacity building, and that hinder sustainability of control and elimination measures, and which will be more extensively described in the following reviews.

**Box 3. Gaps That Hinder Sustainability of Control and Elimination Measures**

Despite the many control initiatives that have been put in place, helminthiases still persist as a public health problem. To facilitate progress towards their control and elimination, it will be necessary to support research and development of the following, which are required for interventions to remain sustainable and thus bring long-lasting benefits to the global public health community:

- Promote a better understanding and appreciation of the importance of human helminthiases as causes of ill-health and extreme poverty, so as to increase allocation of resources for combating such infections, and, in particular, to invest in a continuous effort to improve and update knowledge of helminth fundamental biology and to translate such knowledge into intervention tools
- Optimise existing intervention tools such as improved formulation/combination of treatment regimens with current/novel anthelmintics, control of vectors/intermediate hosts, as well as development of new pharmaceuticals and vaccines
- Develop new diagnostic tools for determination and quantification of: a) infection prevalence and intensity that can aid more accurate mapping of helminth infections; b) intervention impact in monitoring & evaluation (M&E) protocols; c) possible anthelmintic resistance; d) treatment end points
- Develop mathematical and statistical models for understanding parasite epidemiology; refining epidemiological mapping; designing and implementing M&E and surveillance of anthelmintic intervention; and determining criteria for safe cessation of MDA and post-control surveillance
- Conduct research on the environmental and social ecology of human helminthiases in order to improve their control on a large scale and at sustainable levels
- Promote a better understanding of the role basic research can play to facilitate knowledge and the development of improved tools to further support control and elimination measures, and thus bridge the gap between basic research of helminth infections and operational needs
